# Stabilizing magnetic skyrmions in constricted nanowires

**DOI:** 10.1038/s41598-022-14345-0

**Published:** 2022-06-16

**Authors:** Warda Al Saidi, Rachid Sbiaa

**Affiliations:** Department of Physics, Sultan Qaboos University, PC 123, P.O. Box 36, Muscat, Oman

**Keywords:** Materials science, Nanoscience and technology, Physics

## Abstract

Magnetic skyrmions are topologically-protected chiral nano-scale spin structures that offer low power and high-density functionalities for spintronic devices. They behave as particles that can be moved, created and annihilated. These characteristics make them promising information-carrying bits, hence a precise control of the skyrmion motion is essential. This study shows that stabilizing skyrmion is possible using a stepped nanowire geometry. The nanoconstriction dimension and materials properties are found to strongly affect the pinning, depinning and annihilation of the skyrmion. It is also observed that near the stepped region, the skyrmion slows down and its velocity changes direction before its stability. Moreover, a reduction of skyrmion size as it squeezes through the stepped region is observed. Our results will open a new strategy for the design and development of skyrmion-based devices.

## Introduction

In the field of spintronics and magnetic memory devices, the reversal of magnetization by either a magnetic field or an electric current was the basis of changing the magnetic state. Driven by the need for higher storage density, there was a shift to control the magnetic domain wall (DW) rather than the magnetization. Intensive studies were conducted on DW-based devices^1–13^ but the major challenge is the required high current density (10^11^–10^12^ A/m^2^) to move the DW; i.e. high writing current. In addition, the unavoidable defects within the devices lead to an instability issue that can be revealed in a drastic change in the pinning strength. More recently, particle-like magnetic skyrmions^14,15^ attracted much attention due to their nanometer size, their stable spin texture and low power-driven motion with a current density within 10^6^ to 10^10^ A/m^2^^16–27^. Manipulation of skyrmions can be achieved using electric current^16,17,19^, spin acoustic waves^25^ or local field gradient^24^. Although skyrmions can be magnetically stable for materials with a strong Dzyaloshinskii-Morita interaction (DMI)^28,29^, controlling their positions and dynamics remains an objective for their implementation in functional devices. It has been reported that creating semicircular notches along the edges of the nanowire could pin the skyrmions but their topological stability can be lost and can be annihilated due to their interaction with the notches^30^.

In this study, a stepped nanowire is proposed to stabilize magnetic skyrmion. The interaction of the skyrmion with the stepped barrier is investigated for different dimensions and material intrinsic properties. For specific conditions, the skyrmion can be pinned, depinned and even annihilated. The skyrmion size and its dynamics were also found to depend strongly on the device shape and materials properties.

## Results

In this calculation, the nanowire length *L*, width *W* and thickness *t*_z_ were fixed to 300 nm, 75 nm and 3 nm, respectively. The device investigated was discretized into tetragonal cells with a size of 2 × 2 × 3 nm^3^ without considering periodic boundary conditions. The stepped region is added to the nanowire with the objective to act as a pinning site for stabilizing and controlling the motion of the skyrmions position. The intrinsic magnetic parameters used in this study are saturation magnetization *M*_S_ = 500 kA/m, the exchange stiffness *A* = 15 pJ/m, the perpendicular magnetic anisotropy *K*_u_ = 0.8 MJ/m^3^, the damping constant *α* = 0.1 and DMI strength *D* = 3.3 mJ/m^2^. These material properties are considered uniform and are not changed unless otherwise specified. It is worthy to note that these values, except for the damping constant, have been adopted in other studies^32–34^. For the case of materials with perpendicular magnetic anisotropy, *α* around 0.1 is a common value as reported in our previous work^35^. This finite-difference-based micromagnetic software package is developed for our micromagnetic simulation. As initial state, a Néel skyrmion is created at the center of the left side of the nano track with a topological number *Q* =  + 1 and core polarization equal to + 1. This latter value describes the orientation of magnetic moments at the center with respect to the *z*-direction, where the magnetization of the core points in the positive *z*-direction. These parameters lead to a skyrmion that has an intrinsic diameter of around 23 nm, which is determined by the material properties and the confined geometry at *t* = 0.

One of the main objectives of this study is to compare the skyrmion dynamics under a pulsed current in conventional and stepped geometries. The electric current was applied in the form of a rectangular pulse flowing from the left edge along the direction of the wire, which is defined as the *x*-axis so the electrons would flow toward the right. We consider the in-plane current flowing directly through the ferromagnetic material. For such a case, the parameter *β* representing the strength of the nonadiabatic STT was fixed to 0.2, the spin polarization *P* to 0.4 and Slonczewski’s parameter *Λ* as 1^36–38^.

The skyrmion Hall angle *φ*, which indicates the skyrmion transverse motion on the nano-track caused by the Hall effect, is defined as^39^:

1 $$\varphi = ta{n}^{-1} \left(\frac{{v}_{\mathrm{y}}}{{v}_{\mathrm{x}}}\right) = ta{n}^{-1}\left(\frac{\alpha \beta }{\alpha \beta +1}\right)$$

Based on Eq. (1), the skyrmion Hall angle for our material was found to be *φ* = 1.12° indicating a transverse motion of the skyrmion toward the upper edge. The parameters *v*_x_ and *v*_y_ are the *x* and *y* components of the skyrmion velocity.

## Nanoconstriction dimension and skyrmions dynamics

**Figure 1:**
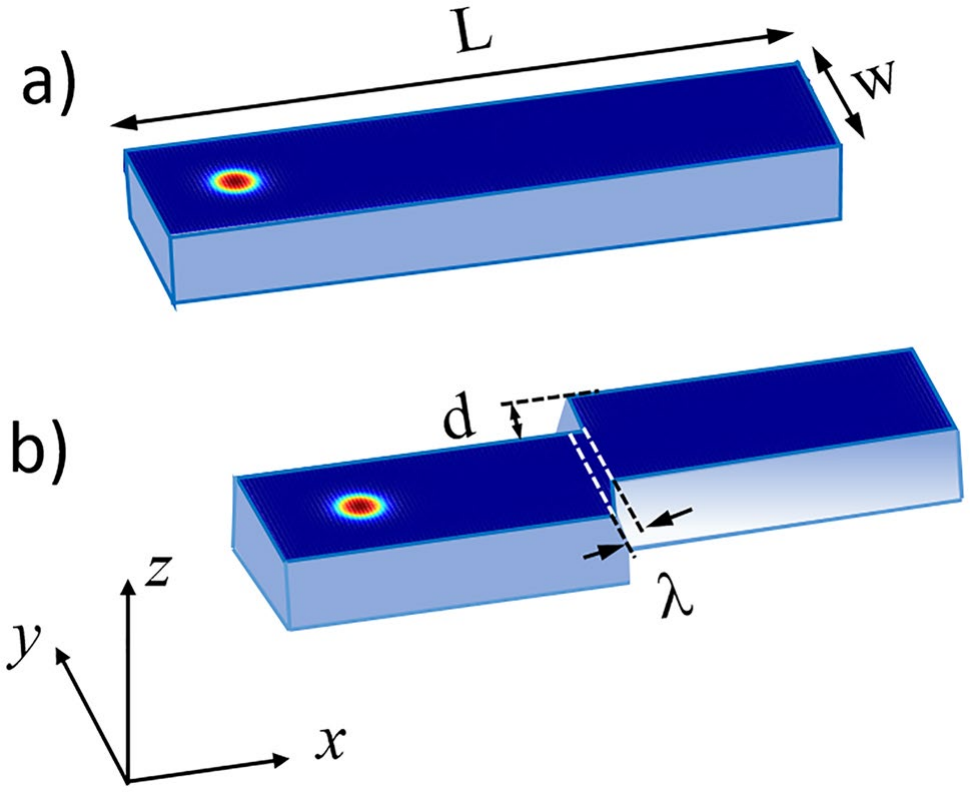
(**a**) and (**b**) are schematic drawings of the conventional and stepped nanowires investigated. The length *L* and the width *W* were fixed to 300 nm, 75 nm, respectively. The current is considered flowing along the *x*-axis.

Skyrmions show inertia-driven drift shortly after the current pulse is removed as reported by Morshed et al.^40^. For memory devices application, such undesirable motion represents a challenge for controlling the position of the skyrmion (magnetic state) within the nanowire. In this study, we investigated the effect of stepped nanowire for controlling the position and the dynamics of skyrmions. To understand the motion of the skyrmion near the stepped region, both the device geometry and the material intrinsic properties were evaluated. The nanoconstrictions were created by varying the parameters *d* and *λ* defining the stepped region geometry as can schematically be represented in Fig. 1. The strength of the pinning and depinning was investigated by varying the material properties *D*, *K*_u_, *M*_S_, *A* and *α*. One parameter was varied at a time while the others were kept constant.

**Figure 2:**
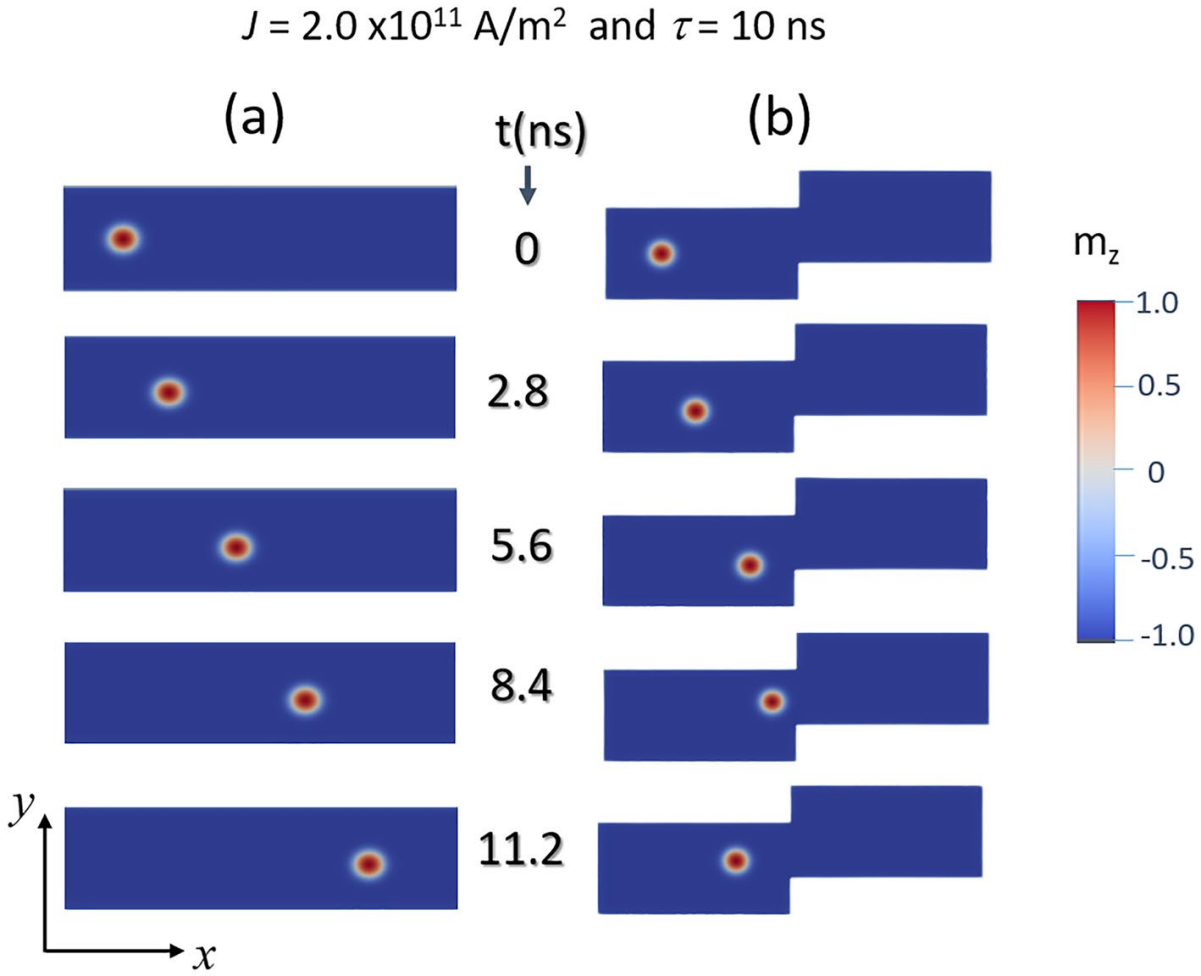
The time dependence of schematic of a skyrmion position for (**a**) conventional nanowire and (**b**) stepped nanowire. The colors represent the *z*-components of the spins. At the core of the skyrmion, the spin is pointing up and at the circumference, the spins are pointing down. The length of the device is *L* = 300 nm, the width *W* = 75 nm and thickness *t*_z_ = 3 nm. The skyrmion is moving along the *x*-axis (from left to right).

**Figure 3:**
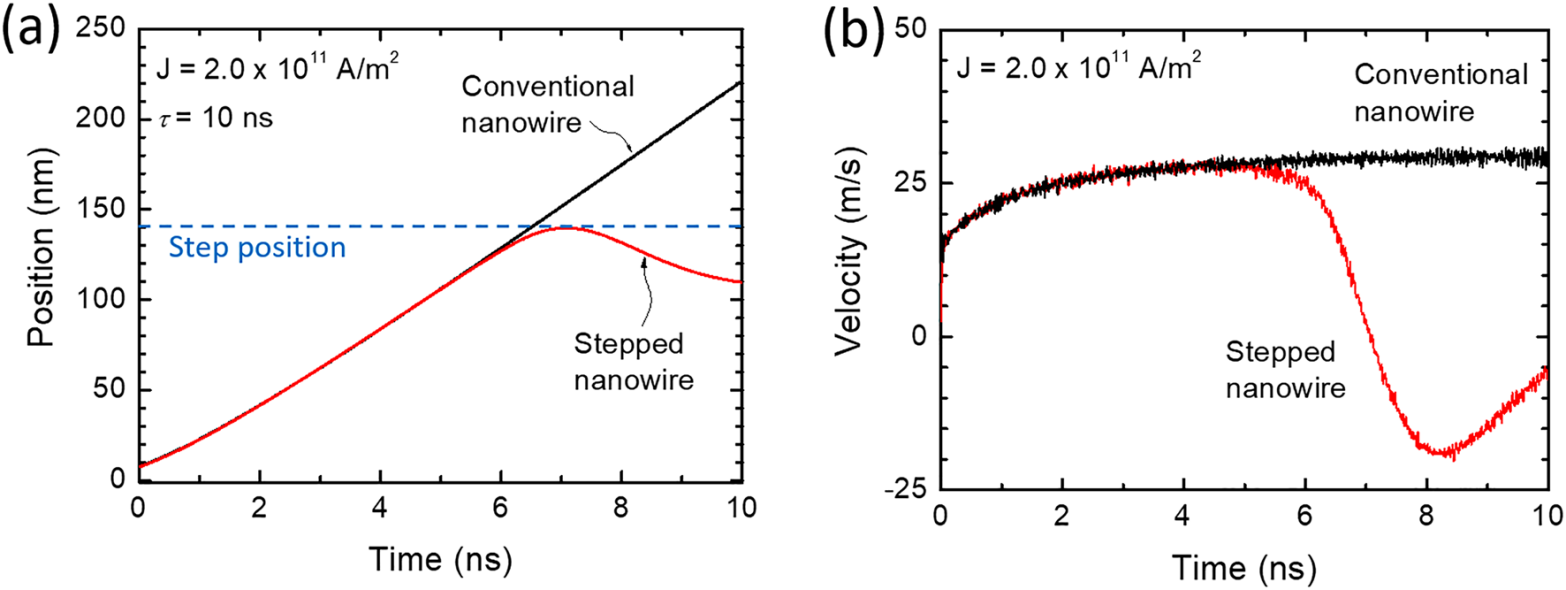
(**a**) The skyrmion position versus time when the current pulse of 2 × 10^11^ A/m^2^ is applied for 10 ns. (**b**) The velocity versus time for the both conventional and stepped nanowires.

Figure 2 shows snapshots of the magnetic skyrmion within a conventional and a stepped nanowire. Each row represents the motion of an isolated skyrmion along the wire for different times using a pulsed current with a fixed amplitude and width. The current density *J* and pulse width *τ* were kept constant at 2.0 × 10^11^ A/m^2^ and 10 ns, respectively. Firstly, the simulation was conducted on a conventional nanowire to investigate the dynamics of the skyrmion (Fig. 2a). As can be seen from this figure, the skyrmion moves continuously from the left edge to the right one along the *x*-axis. There is an almost linear time dependence of the skyrmion position with a maximum velocity of about 25 m/s as plotted in Fig. 3. In contrast to the conventional nanowire, the stepped design shows that the skyrmion is sensitive to the step in the wire (Fig. 2b). It can be noticed that for *t* ~ 5.6 ns, the skyrmion slows down until reaches the stepped region (*t* around 8.4 ns) then it slowly bounces back. The applied current has been chosen to be below a critical value that drives the skyrmion beyond the stepped region as will be discussed later. The color scale represents the magnetic moments’ components in the *z*-direction (out-off plane). A comparison of the time dependence of the position of the skyrmion for the cases of conventional and stepped nanowires is plotted in Fig. 3a. It can be seen that at around 6 ns, the skyrmion is near the middle then bounces back and gets stabilized at a position of 110 nm from the edge after 11 ns. The behavior of the velocity with time is plotted in Fig. 3b revealing a negative value in the time range between 7 and 8 ns (skyrmion changes direction) before moving forward until reaching the physical barrier (step) and stabilized. When the current pulse is applied for a duration of 10 ns, the total energy, anisotropy energy, and demagnetization energy significantly oscillate during this period due to the rotation of magnetic moments of the skyrmion structure. Once the current is removed, no oscillations are observed as has also been reported in Ref.^41^. When the skyrmion reaches the stepped region, the demagnetization energy shows a sharp increase. It is worthy to notice from Fig. 3b that in the time range between 2 and 6 ns, the skyrmion reaches a maximum speed of ~ 25 m/s then when it is closer to the physical barrier (*t* ~ 8.4 ns), it slows down (negative velocity) for about 2 ns with a maximum speed of around 20 m/s. Finally, the skyrmion is stabilized closer to the stepped region without being in contact.

**Figure 4:**
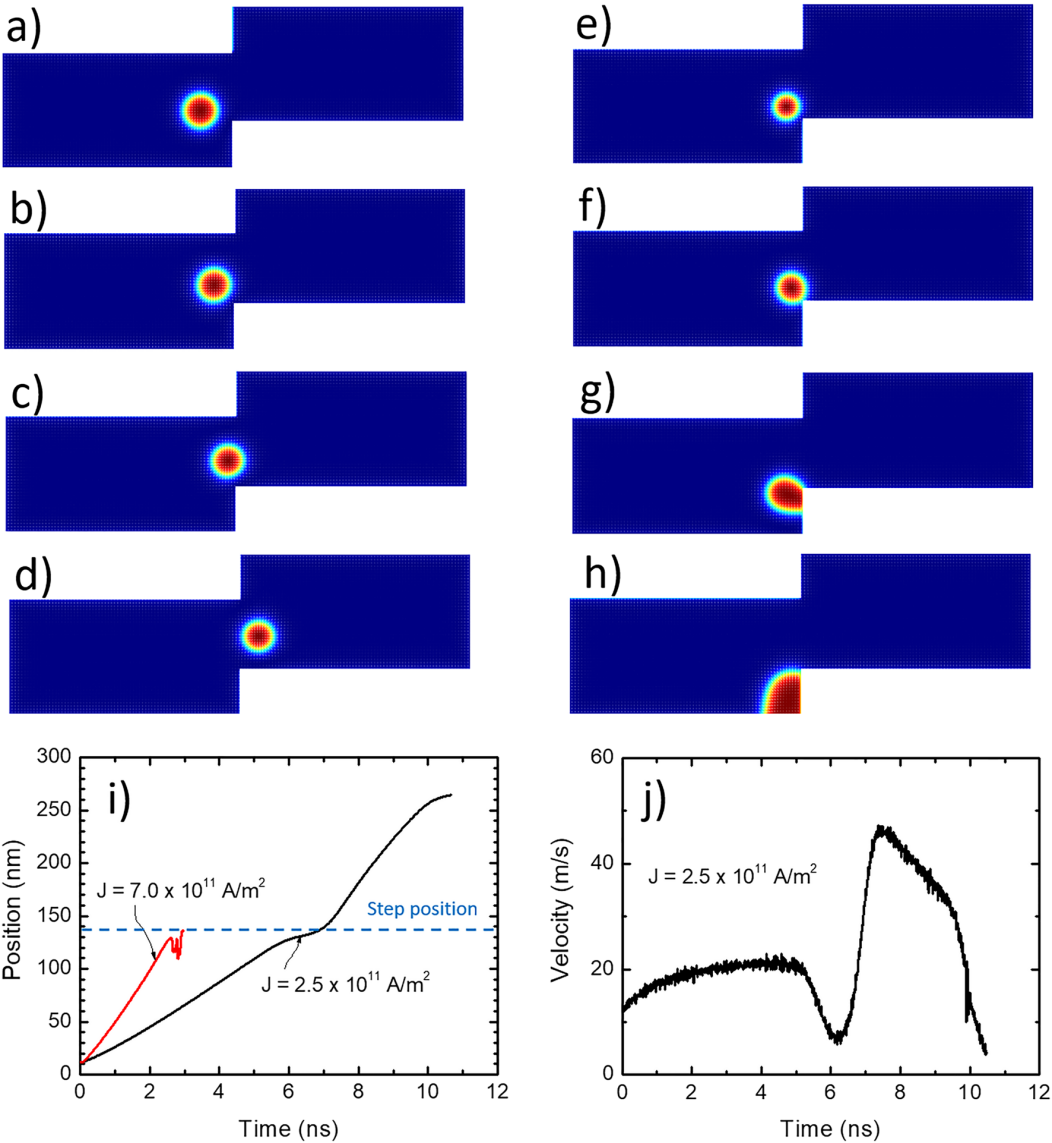
The motion of skyrmion within a stepped nanowire for (**a**–**d**) *J* = 2.5 × 10^11^ A/m^2^ where the skyrmion could overcome the stepped region and (**e**–**h**) *J* = 7.0 × 10^11^ A/m^2^ where it was annihilated. The snapshots (**a**) to (**d**) are taken for *D* = 3.55 mJ/m^2^ at *t* = 2.61 ns, 2.79 ns, 3.06 ns and 3.24 ns, respectively while images taken for (**e**–**h**) are for *t* = 2.34 ns, 2.43 ns, 2.52 ns and 2.61 ns (*D* = 3.30 mJ/m^2^), respectively. (**i**) is a plot of the time dependence of the skyrmion position for *J* = 2.5 × 10^11^ A/m^2^ and 7.0 × 10^11^ A/m^2^ and (**j**) is the velocity for the skyrmion moving under *J* = 2.5 × 10^11^ A/m^2^.

To investigate the depinning process, the stabilized position near the step is considered as the initial state and then a second pulse with varied magnitude and width is applied until the skyrmion is depinned. Depending on the current density, the skyrmion shows different behaviours as shown in Fig. 4. For current density below a threshold value, the skyrmion stops when it reaches the stepped region. The critical current density of the depinning (*J*_c_) was investigated for *d* = 30 nm at *λ* = 0. At larger *J* values, the skyrmion is released from the stepped region (depins) and continues its motion. It was observed that its velocity increases sharply when it passes through the stepped region. The value of *J*_c_ strongly depends on the size of the skyrmion and the geometry of the device. For the values of *d* and *λ* discussed earlier, it was found that the minimum *J* to move the skyrmion from the stepped site is 2.4 × 10^11^ A/m^2^, where the skyrmion can overcome the energy barrier and pass over the stepped region. However, if *J* is smaller than this critical value, the skyrmion will not be able to overcome the barrier even if the pulse width is increased. By analyzing the motion of the skyrmion at low current (*J* < *J*_c_), we noticed that the skyrmion moves around its own pinning position before the pinning occurs. For *J* > *J*_c_, the skyrmion passes the stepped region without annihilation until reaching the end of the nanowire as can be seen in Fig. 4a–d for *D* = 3.55 mJ/m^2^ where the skyrmion could be seen during its movement near the stepped area. Interestingly, a small reduction of the skyrmion size was observed during this passage before recovering until reaching the end of the device or closer depending on the current magnitude and pulse width. Figure 4i shows the time dependence of the skyrmion position for two different values of *J*. For *J* = 2.5 × 10^11^ A/m^2^ (*D* = 3.30 mJ/m^2^) continuous displacement of the skyrmion under a pulsed current can be seen with a distinguished three phases. In the time range *t* < 6.2 ns, the skyrmion was moving linearly with time at a constant velocity of ~ 20.5 m/s until being stabilized a few nanometers from the edge of the step. For about 1 ns (6.2 ns–7.1 ns), the velocity of the skyrmion drops by about half (~ 11 m/s) before overcoming the stepped region and gaining momentum with a velocity of ~ 33.5 m/s. For relatively large values of current density (*J* > 7.0 × 10^11^ A/m^2^ and *D* = 3.30 mJ/m^2^) as shown in Fig. 4e–h, the skyrmion is moving with a larger velocity of ~ 50 m/s since the current density is high. The velocity of the skyrmion under *J* = 2.5 × 10^11^ A/m^2^ is shown in Fig. 4j. However, the skyrmion gets annihilated at the edge of the stepped region which puts an upper limit on the skyrmion operating current. The images shown in Fig. 4e–h were taken when the skyrmion is near the step region showing the annihilation process.

By changing the nanoconstriction size, it was found that the critical current density increases with increasing the depth of step *d*, which was also reported in the case of notches^30^. For actual memory applications, the pinning strength due to the nanoconstriction should be high enough to guarantee good stability of the skyrmion but not excessively high so that the depinning (change of the magnetic state) can occur at an acceptable current magnitude. This feature is important for the application of the skyrmion-based magnetic racetrack because a smaller current density of depinning means less energy consumption and less Joule heating induced by the current. The value of *d* was varied while the other parameters remained constant. It was observed that the strength of the pinning and depinning of the skyrmion is strongly dependent on the step dimension.

**Figure 5:**
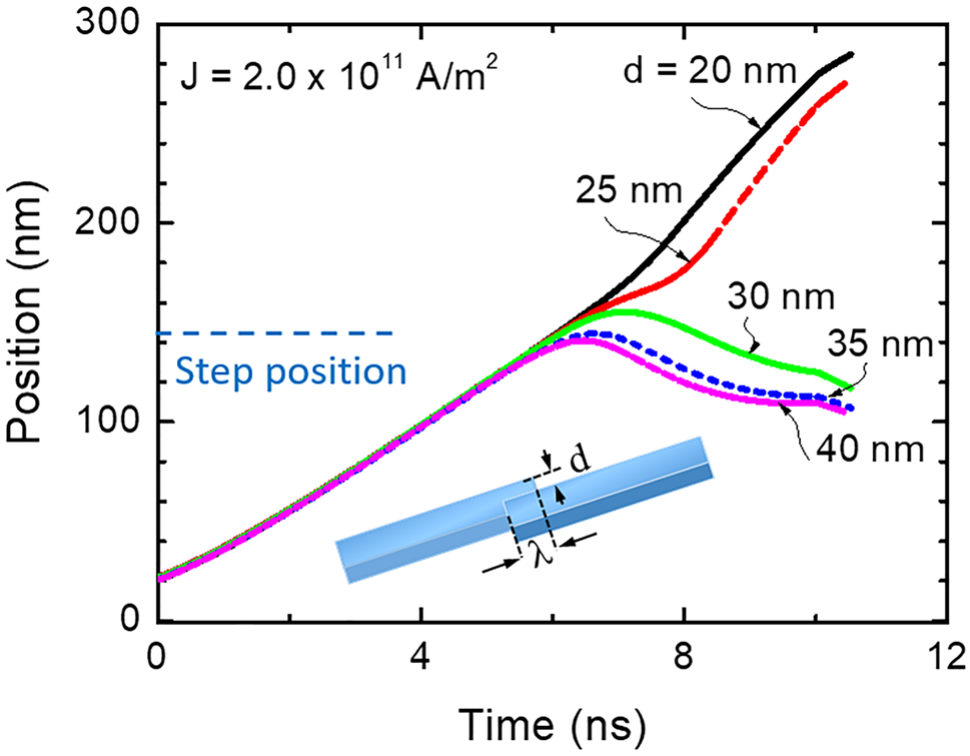
The position of the skyrmion versus time for *λ* = 0 and different values of *d*.

**Figure 6:**
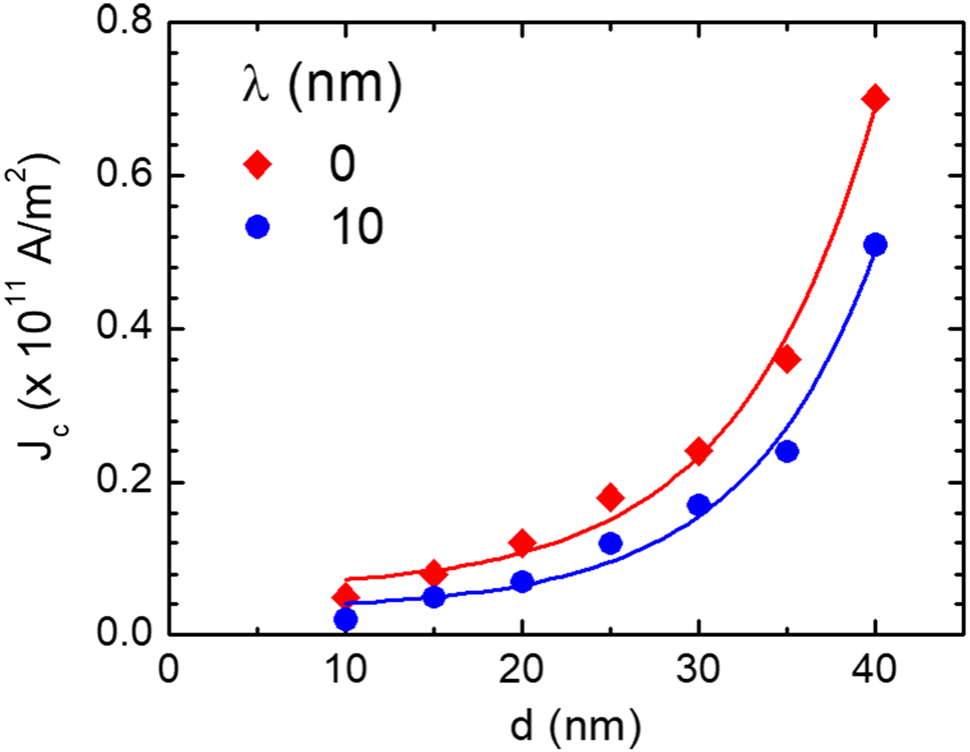
The critical or minimum current density for depinning a skyrmion versus the depth *d* of the stepped region.

Under the conditions discussed above and for a current density of 2.0 × 10^11^ A/m^2^, the skyrmion was not able to overcome the physical barrier for *d* = 30 nm and *λ* = 0 nm. Insert of Fig. 5 shows the schematic of the racetrack with stepped geometry used for the simulation. We create the stepped region by varying the dimensions of both *d* and *λ* as has been reported in our previous work to stabilize the magnetic domain wall^42^. The dynamics of a skyrmion for different values of *d* is shown in Fig. 5. The pinning was not possible for smaller values of *d* but even the skyrmion was not pinned, its energy was affected; i.e., the energy decreased with *d* and its velocity became smaller. The critical (minimum) current density *J*_c_ for depinning the skyrmion from the stepped region decreased by increasing *λ*. The geometry of the stepped region provides an easy way to adjust the pinning strength for skyrmion; by enlarging the size of *λ*, the skyrmion can be more easily released. When the pinning site dimension varies while other parameters are constant, the pulse width needed to pin and depin the skyrmion has to be optimized depending on the position of the stepped region. The current density *J*_c_ was investigated as a function of *d* and for two values of *λ* as can be seen in Fig. 6. The *J*_c_ shows an exponential increase with *d* for both values of *λ*. It is thus possible to tune the current density for pinning and depinning the skyrmion by controlling the stepped region dimension.

### Materials properties and skyrmions dynamics

**Figure 7:**
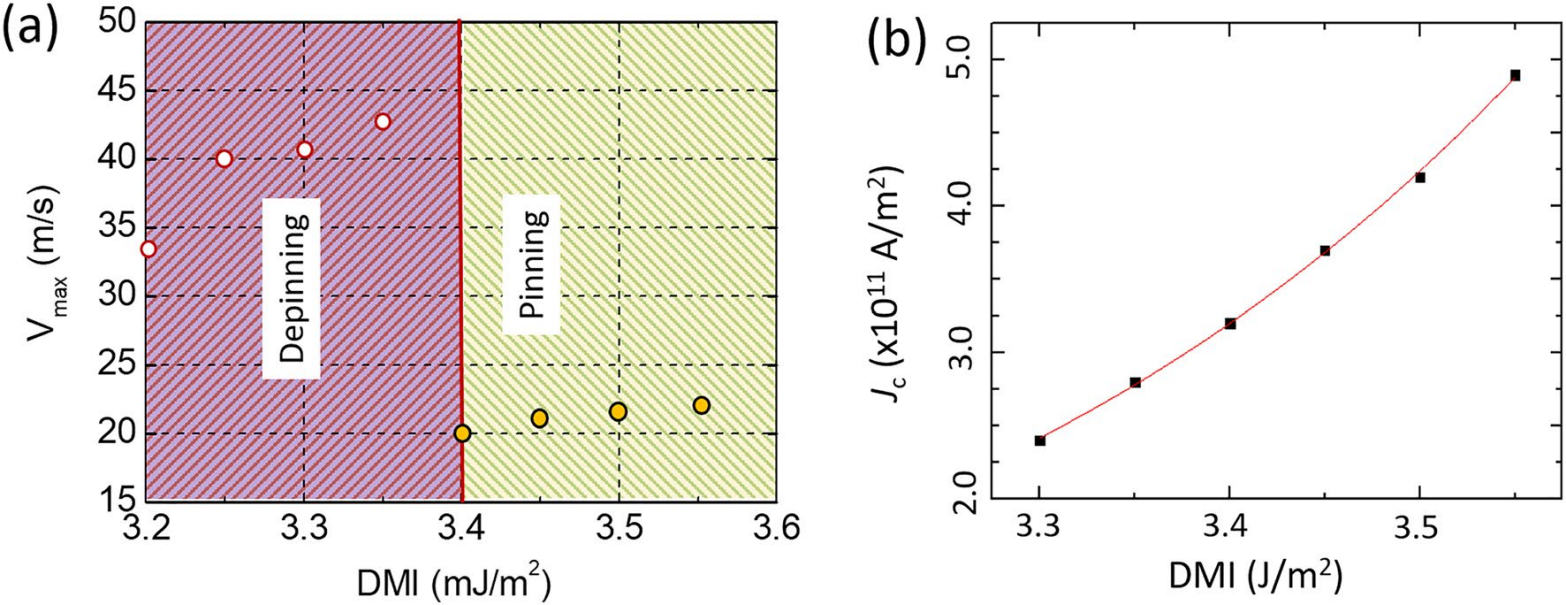
(**a**) The maximum velocity of the skyrmion in a stepped nanowire versus DMI. High DMI favours the pinning of the skyrmion while it is easier to release it from the stepped region at lower DMI. In both cases, the velocity is continuously increasing with DMI. (**b**) The dependence of the critical on DMI which shows exponential growth.

**Figure 8:**
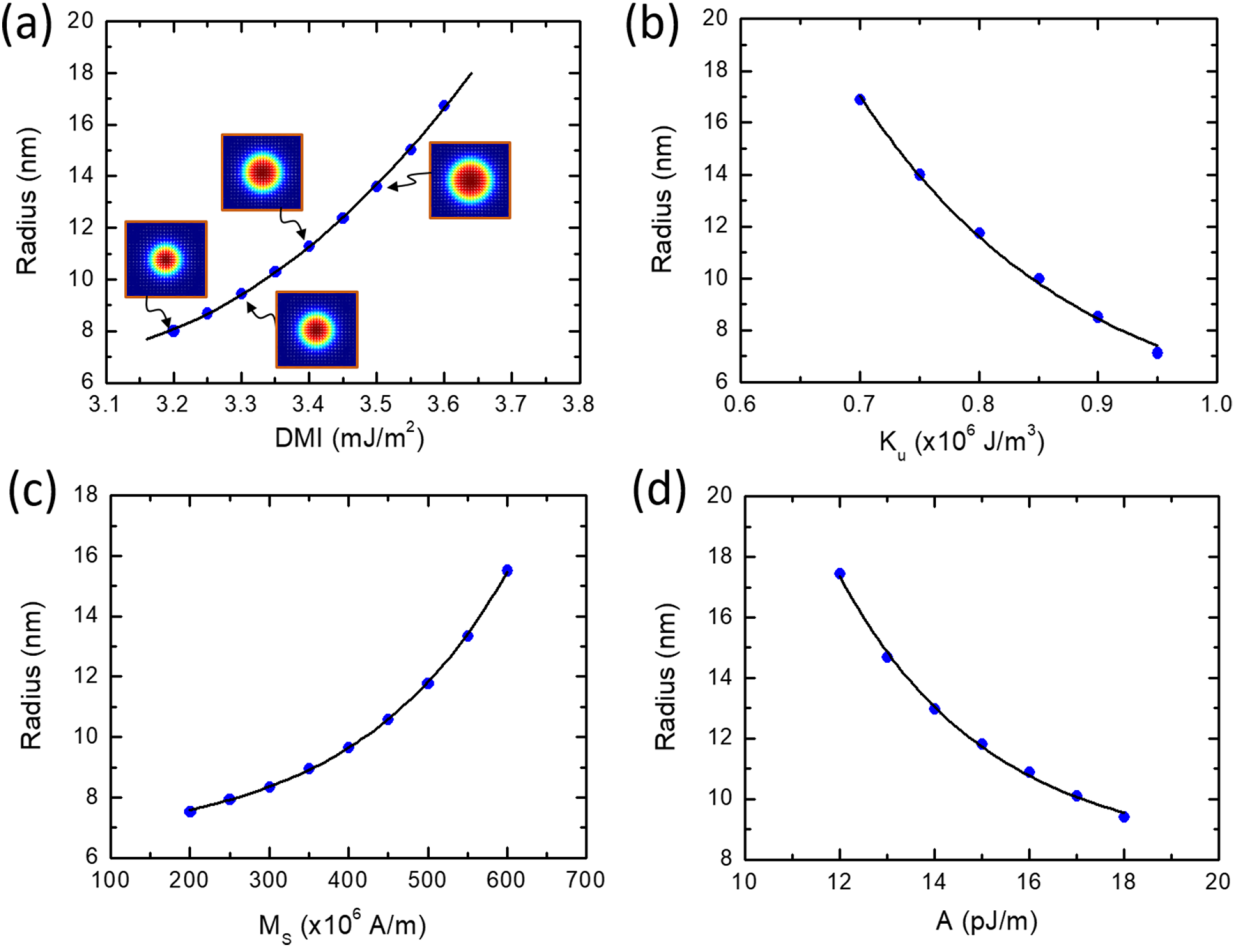
The dependence of the skyrmion radius on (**a**) DMI, (**b**) magnetic uniaxial anisotropy energy, (**c**) saturation magnetization and (**d**) exchange stiffness. Case (**a**) reveals a second-order polynomial growth function, (**b**) and (**d**) show an exponential decay of the radius with *K*_u_ and *A* while for (**c**) the radius grows exponentially with *M*_s_.

In this part of the study, the focus will be on material properties in controlling the skyrmion dynamics. One of the key elements for stabilizing the magnetic skyrmions is the DMI strength, which originates from the breaking of the bulk inversion symmetry. Changing the DMI values offers another way for controlling the size of the skyrmion. A decrease in the total energy with the DMI which is due to lowering the DMI energy was observed. For a fixed DMI value, the total energy drops when reaching the edge of the device. From Fig. 7a, it can be seen that a larger skyrmion moves faster which is desirable for faster data transfer. The data in Fig. 7 is for *J* = 2.0 × 10^11^ A/m^2^ and *τ* = 8 ns, respectively. At *J* = 3.0 × 10^11^ A/m^2^, the skyrmion is pinned for a minimum DMI of 3.4 mJ/m^2^. In both regions, the speed of the skyrmion is increasing with DMI. As the DMI increases, the size skyrmion becomes larger as can be seen in Fig. 8a. An increase of DMI from 3.2 to 3.6 mJ/m^2^ leads to an expansion of the skyrmion. The critical DMI is calculated using the following equation ${D}_{crit}=4\sqrt{A ({K}_{u}-0.5{\mu }_{o}{M}_{s}^{2})}/\pi$ and is found to be 3.78 mJ/m^2^^43,44^. As the size of the skyrmion expands with DMI, it becomes difficult to cross the stepped region as a result of a high energy barrier. Because of the increase of the skyrmion size with DMI, *J*_c_ shows also the same trend as discussed in Fig. 6. For instance, a change of DMI from 3.2 to 3.5 mJ/m^2^ leads to about 75% increase of the skyrmion radius (from 9.4 nm to 13.6 nm) and consequently the minimum current density to depin it needs to be increased by almost the same amount (71%) as shown in Fig. 8a. The critical current was found to increase exponentially with increasing DMI (Fig. 7b).

In contrast to DMI, the skyrmion shrinks by increasing *K*_u_ as can be seen in Fig. 8b. The critical current density *J*_c_ required to move the skyrmion increases because of increasing the anisotropy. As expected, a faster motion of the skyrmion can be achieved with a lower *K*_u_.

Figure 8c is a plot of the change of the skyrmion radius with *M*_S_. The skyrmion size expands with *M*_S_ following an exponential behavior *R* = ζ *exp*(*M*_S_/*M*_O_); however, the increase of the radius *R* with DMI is following a second-order power function. The exchange stiffness *A* is another key material parameter that has been investigated to understand the skyrmion dynamics. The skyrmion becomes small as *A* increases. For values between 12 pJ/m and 18 pJ/m, the radius was found to decrease exponentially from 17.45 nm to 9.42 nm (Fig. 8d). Based on our device with *d* = 30 nm, *λ* = 0 nm and *J* = 3.0 × 10^11^ A/m^2^, the skyrmion is able to pass through the barrier only for *A* above 14 pJ/m. For materials with lower values of *A*, a larger current density is required. A larger speed can be achieved for the skyrmion in the nanotrack with smaller exchange stiffness *A*. In such a case, the spins around the skyrmion are easy to reverse, leading to a larger velocity^45^.

## Discussion

It has been demonstrated that skyrmions can be stabilized in stepped magnetic nanowires. Their dynamics depend strongly on the nanoconstriction size, the current density and the material properties. This variety of parameters provides an easy way to control the pinning and depinning of the skyrmion. For large current density, the study showed that it is possible to annihilate the skyrmion through a collision with the edge of the step. Interestingly, and for low current density values, the pinning of the skyrmion occurs by a reduction of its velocity followed by a slight repulsion from the step before final stability. The release of the skyrmion from the vicinity of the stepped region is possible by a slight increase in the current density. The value of the minimum current to depin the skyrmion is easily controllable by the material properties such as *M*_S_, *K*_u_, DMI and *A*. It is thus possible to create a multi-state device based on skyrmion for neuromorphic computing and large capacity memory.

## Methods

The simulation is performed using MuMax3, a graphical processing unit (GPU) accelerated micromagnetic simulation^31^. The main equation to be solved to study the stability and the dynamical response of a single Néel skyrmion is based on the Landau-Lifshitz-Gilbert (LLG) formalism that describes the time evolution of the magnetization:

2 $$d{\varvec{M}}/dt=-{\gamma }_{o}{\varvec{M}}\times {{\varvec{H}}}_{\mathrm{eff}}+ \frac{\alpha }{{M}_{\mathrm{s}}} ( M\times d{\varvec{M}}/dt)+ \frac{u}{{{M}_{\mathrm{s}}}^{2}} / \left[{\varvec{M}}\times \left(\frac{\partial {\varvec{M}}}{\partial x}\right)\times {\varvec{M}}\right] - \frac{\beta u}{{M}_{\mathrm{s}}}\left({\varvec{M}} \times \frac{\partial {\varvec{M}}}{\partial x}\right)$$

where ***M*** is the magnetization vector, *t* is the time, *γ*_o_ is the gyromagnetic ratio, ***H***_**eff**_ is the effective magnetic field, *α* is the Gilbert damping coefficient and *M*_**S**_ is the saturation magnetization. Equation (2) is for the case where the current is flowing in the plane of the device. The first term in the right side of the equation, known as the Larmor precession, describes the precession movement that the magnetic moments perform around the effective magnetic field when they are not completely aligned. During this precession, the magnetization relaxes along the direction of the field until becomes aligned with it to minimize the energy of the system that is modeled by the second term containing the Gilbert damping. The last term in Eq. (2) is for the current-in-plane where *u* represents the adiabatic STT coefficient and *β* being the strength of the nonadiabatic STT.

The effective field applied on the unit cell is defined by the expression

3 $${H}_{\mathrm{eff}}= -\frac{1}{{\mu }_{o}}\frac{\partial E}{\partial M}$$

where ${\mu }_{o}$ the permeability free space, *E* the average energy density which contains includes the magnetic anisotropy energy, the exchange energy, DMI and the magnetostatic energy.

## Supplementary Information

Supplementary Information 1.

Supplementary Video 1.

## Data Availability

All data generated or analysed during this study are included in this published article and its Supplementary Information files.
