# Supplementary material for: Stabilizing magnetic skyrmions in constricted nanowires
Source: Sci Rep. 2022 Jun 16;12:10141. doi: 10.1038/s41598-022-14345-0 (PMC9203817; doi:10.1038/s41598-022-14345-0)
Supplement: Supplementary file 1 — Supplementary Information 1. [file 41598_2022_14345_MOESM1_ESM.pdf]

Stabilizing magnetic skyrmions in constricted nanowires

Warda Al Said[1] and Rachid Sbiaa[1]*

[1] Department of Physics, Sultan Qaboos University, P.O. Box 36, PC 123, Muscat, Oman

***Correspondence to rachid@squ.edu.om**

Supplementary Information

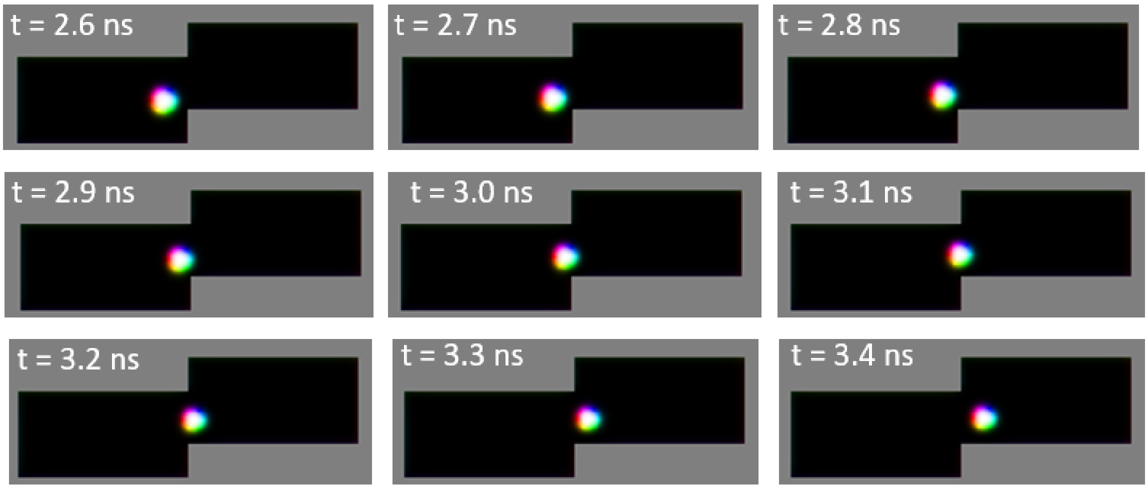

Figure S1. Snapshots of skyrmions within a stepped nanowire. For different calculation times.
The current density was fixed to $J = 2.5 \times 10^{11}$ A/m^2. The material properties are M_S = 500 kA/m, A = 15 pJ/m, K_u = 0.8 MJ/m^3, α = 0.1 and D = 3.3 mJ/m^2.

Supplementary Movie Captions

Supplementary Movie 1. The motion of a skyrmion within a stepped nanowire for current density of 7.0×10^{11} A/m^2 . The material properties are The material properties are M_S = 500 kA/m, A = 15 pJ/m, K_u = 0.8 MJ/m^3, α = 0.1 and D = 3.3 mJ/m^2.
